# Supplementary material for: Complexome profiling of the Chlamydomonas psb28 mutant reveals TEF5 as an early PSII assembly factor
Source: Plant Cell. 2025 Mar 17;37(6):koaf055. doi: 10.1093/plcell/koaf055 (PMC12164590; doi:10.1093/plcell/koaf055)
Supplement: koaf055_Supplementary_Data [file koaf055_supplementary_data.zip › psb28_interactive.zip!/plots/intensity/qualityPlots/peptides/CPL10_Cre04.g228450.t1.2.html]

### Cre04.g228450.t1.2

Trivial name: CPL10  
SeqCoverage [median]: 13.60  
Mass: 29.73 kDa  
  
 

| Sequence | MissedCleavage | Mass [Da] | wtIntensity | muIntensity | Score |
| --- | --- | --- | --- | --- | --- |
| KLNFDD | 1 | 750.35 | 3459.28 | 4313.75 | 140.01 |
| KYTQAFKDDIGDK | 2 | 1527.76 | 290.62 | 0.00 | 58.07 |
| LPQEQQQELLR | 0 | 1380.74 | 6977.40 | 15685.24 | 221.59 |
| SIFEMQKLPQEQQQELLR | 1 | 2244.16 | 738.84 | 2348.52 | 99.45 |
| YTQAFKDDIGDK | 1 | 1399.66 | 446.62 | 0.00 | 48.00 |
|  |  |  | 11912.8 | 22347.5 |  |
